# Reframing the starting point of shared decision-making under algorithmic short-form video exposure: bone tumor care as a sentinel context

**DOI:** 10.3389/fpubh.2026.1829734

**Published:** 2026-08-24

**Authors:** Jia-Wen Wang, Ying-Fa Feng, Zi-Bo Zhang

**Affiliations:** Department of Orthopedics, The Fourth Hospital of Hebei Medical University, Shijiazhuang, Hebei, China

**Keywords:** bone metastasis, cognitive anchoring, health misinformation, immune checkpoint inhibitors, medication-related osteonecrosis of the jaw, shared decision-making, short-form video exposure

## Abstract

Short-form video platforms have become a major component of the pre-consultation information environment for patients with cancer in China and may create new challenges for clinical communication. Contemporary shared decision-making (SDM) and patient decision aids (PtDAs) do not explicitly assess or calibrate beliefs formed before consultation. Algorithmic recommendation and emotionally charged narratives may therefore contribute to pre-consultation cognitive anchoring and alter the conditions under which SDM occurs. We use bone tumor care as a sentinel context rather than claiming that these patients are uniquely susceptible to algorithmic influence. Patients with primary bone tumors or bone metastases may receive immune checkpoint inhibitors (ICIs), and some patients with bone metastases also receive bone-modifying agents. Because prevention of medication-related osteonecrosis of the jaw (MRONJ) is most effective before bone-modifying therapy begins, cognition at the initial consultation may be particularly consequential. We hypothesize that pre-consultation anchoring could contribute to delayed reporting of immune-related adverse events (irAEs), gaps in MRONJ prevention, reduced ICI adherence, and more 90-day unplanned visits. The cited studies establish the clinical importance and recognized determinants of these outcomes but do not demonstrate that short-form video exposure causes them. To support future testing, we propose the Digital Exposure-Cognition-Anchoring assessment (DECA) framework: DECA-E records pre-consultation exposure; DECA-C screens and calibrates cognitive anchors; and DECA-L prospectively evaluates longitudinal associations across exposure, cognition, SDM, and clinical safety. DECA is an unvalidated proposal that requires feasibility and outcome evaluation.

## 1 Introduction

Short-form video platforms and other social media have become important sources of medical information for patients with cancer in China. By December 2023, China had 1.053 billion short-form video users, representing 96.4% of the country’s internet users (1). Among patients with gynecologic cancer, platforms such as Douyin and Baidu are frequently used to seek information about disease, treatment, and prognosis after diagnosis (2). However, the overall quality of cancer-related content on Chinese new media is low, particularly for video-based media, and misleading content is common (3). Some patients may therefore arrive at initial or follow-up consultations with misconceptions influenced by prior short-form video exposure, creating potential difficulties for subsequent clinical communication and care.

Contemporary SDM models and PtDAs are widely used to structure communication about patient goals, preferences, options, and risks. However, they do not include a dedicated step for identifying and calibrating pre-existing beliefs repeatedly reinforced by algorithmic information environments (4, 5). Frequent short-form video exposure may contribute to cognitive anchoring before the first consultation across treatment expectations, risk perceptions, and views of physician authority (6, 7). If such anchors remain unrecognized, they may interfere with mutual understanding during SDM and could subsequently affect adherence and cooperation. These proposed effects require direct empirical testing.

Patients with primary bone tumors and those with secondary bone involvement may receive immunotherapy (8, 9). Patients with bone metastases from breast, lung, prostate, and other solid tumors commonly receive bone-modifying agents, and those who meet indications for ICIs may receive both therapies (10, 11). This creates concurrent treatment risks, including MRONJ, which is difficult to treat once established and is best addressed through dental assessment and necessary care before bone-modifying therapy begins (12). We do not argue that patients with bone tumors are uniquely susceptible to algorithmic influence; the anchoring mechanism may apply across oncology populations. Rather, bone tumor and bone metastasis care provides a clinically informative sentinel setting because concurrent ICI and bone-modifying therapy can combine symptom-reporting demands with a time-sensitive preventive window. Short-form video-related anchoring could theoretically affect symptom interpretation and preventive decisions (13). Nevertheless, studies on irAE reporting, MRONJ prevention, ICI adherence, and unplanned care (10, 12, 14–18) establish the importance and recognized determinants of these outcomes; they do not show that short-form video exposure causes them. The exposure-to-outcome links advanced here are hypotheses for future study.

This potential pathway is often framed primarily as a problem of communication skills or health literacy. Interventions limited to communication training and health education may be insufficient if algorithmically reinforced beliefs are already present before consultation (19, 20). A broader public health approach should therefore examine the upstream information environment while retaining established clinical communication and education strategies.

**Figure 1:**
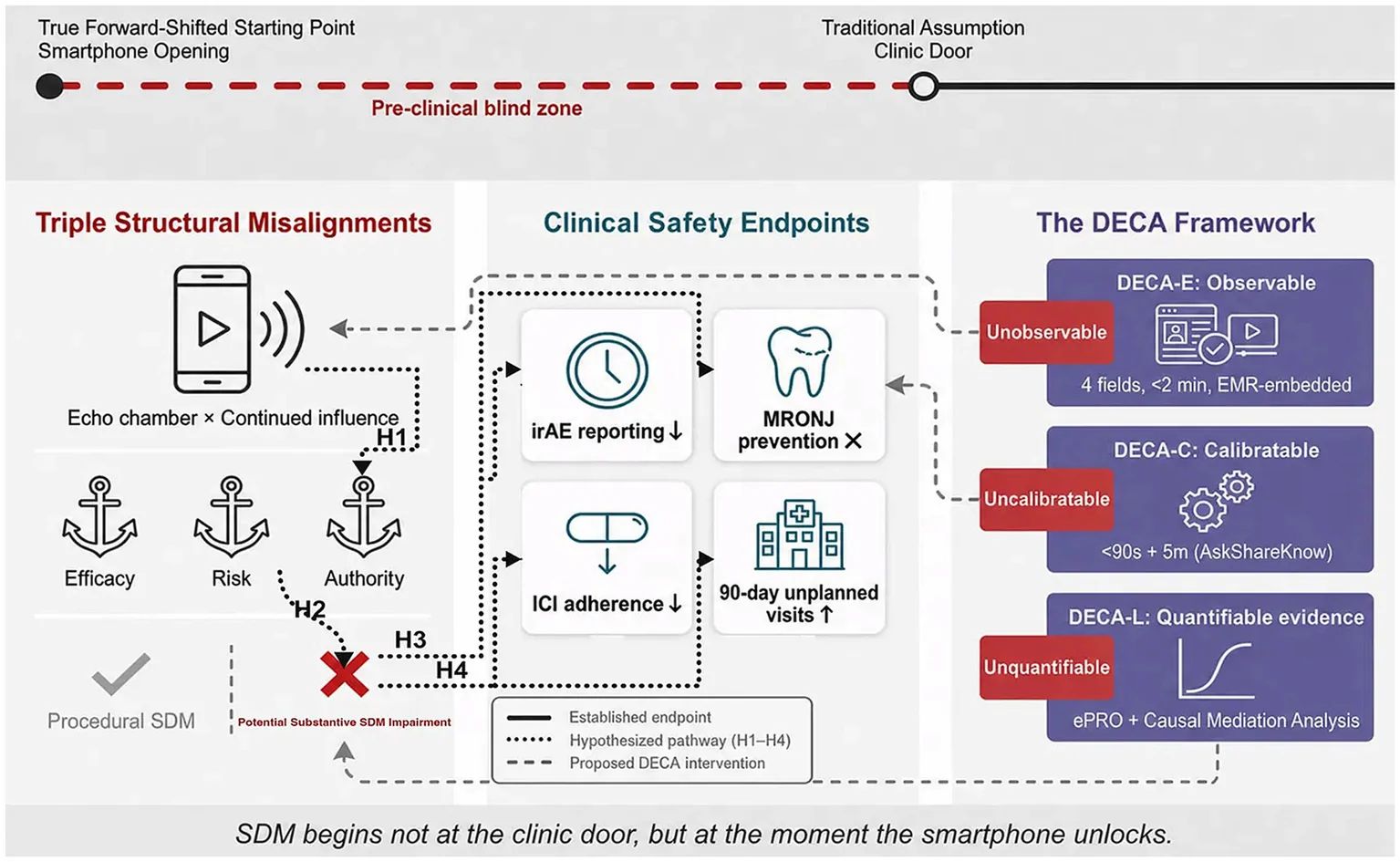
The starting point of shared decision-making (SDM) under algorithmic short-form video exposure: proposed structural misalignments, clinical safety endpoints, and the DECA framework. Bone tumor care is used as a sentinel context. Line types encode evidentiary status. The four clinical endpoints shown as solid cards are established outcomes of documented clinical importance with recognized determinants. Black dotted arrows (H1-H4) denote hypothesized pathways: H1, Algorithmic exposure may contribute to pre-consultation cognitive anchoring across efficacy, risk, and physician-authority expectations; H2, Anchoring may impair substantive SDM even when procedural SDM is completed; H3, Impaired substantive SDM may contribute to delayed irAE reporting and gaps in MRONJ prevention; and H4, It may contribute to reduced ICI adherence and more 90-day unplanned visits. These pathways were not tested in the present work and are not established by the cited evidence; evaluating them is the purpose of the DECA-L proposal. Gray dashed arrows denote proposed DECA interventions rather than demonstrated effects. DECA-C comprises a universal anchor screen lasting less than 90 s, followed by a five-minute calibration script only for patients who screen positive. DECA-C also addresses MRONJ prevention, for which pretreatment dental assessment is the preferred, time-sensitive opportunity, although continued oral care remains necessary after bone-modifying therapy begins.

We propose that, under conditions of repeated algorithmic exposure, the operational starting point of SDM may move upstream from the consultation room to the patient’s pre-consultation use of a smartphone. Three potential mismatches warrant simultaneous investigation: algorithmically amplified exposure may conflict with evidence-based reasoning; existing SDM workflows may not systematically detect reinforced cognitive anchors; and safety in bone tumor and bone metastasis care may be sensitive to misunderstanding because reporting and preventive actions are time dependent. We therefore propose the DECA framework. DECA-E makes pre-consultation exposure observable; DECA-C makes three dimensions of cognitive anchoring assessable and potentially calibratable; and DECA-L is a prospective research design for estimating associations across exposure, cognition, SDM, and clinical safety (Figure 1). The feasibility, measurement properties, and effects of all three modules remain to be established.

## 2 Core mechanisms of pre-consultation cognitive exposure

Before entering the consultation room, some patients may already have encountered repeated short-form video and social media narratives that shape efficacy expectations, risk perceptions, and views of physician authority (21). We hypothesize that effects at the information, decision-paradigm, and clinical levels may accumulate and reinforce one another. This proposed process could create pre-consultation cognitive anchoring relevant to subsequent SDM and to safety-related behaviors such as irAE reporting, MRONJ prevention, and ICI adherence, but the complete pathway has not yet been demonstrated.

At the information level, engagement-optimized recommendation systems can repeatedly deliver homogeneous content and contribute to echo chambers (21). False information can spread farther, faster, and more broadly than accurate information (22), increasing the possibility of repeated exposure to similar misinformation. A systematic review and meta-analysis found that approximately 27% of cancer-related new-media content contained misinformation and a further 12% showed commercial bias (3). Cancer-related videos may therefore be difficult for patients to evaluate reliably (23). This concern is relevant to bone tumors: available videos have shown low overall quality and limited treatment information (24, 25). Low-quality content can nevertheless attract substantial engagement (3), which may further increase its algorithmic visibility. In addition, misinformation may continue to influence judgments even after correction, a phenomenon known as the continued influence effect (26, 27). Together, these mechanisms provide a plausible basis for persistent anchoring, although they do not establish its clinical consequences in the population considered here.

At the SDM level, contemporary frameworks do not include a dedicated procedure for detecting and recalibrating beliefs formed before consultation (4). PtDAs and question prompt lists can improve knowledge and participation (5, 28), but they were not designed specifically to measure algorithmically reinforced anchors. Misunderstanding of treatment goals remains common among patients with advanced cancer (29). CollaboRATE and SDM-Q-9 are only moderately correlated (30), indicating that the instruments capture partly different aspects of SDM; this finding does not itself prove cognitive anchoring or substantive SDM failure. We therefore treat the proposition that SDM may be procedurally completed while substantive understanding remains impaired as a testable hypothesis. Future validation should measure pre-consultation anchoring directly and examine its relationship with SDM-Q-9, CollaboRATE, and decision regret rather than infer this relationship from procedural completion alone.

At the clinical level, established evidence and the hypothesized pathway should be distinguished. irAEs can affect any organ system, and early detection depends partly on patients recognizing and reporting symptoms (14, 15). Electronic symptom monitoring has improved patient-reported outcomes and, in one trial, overall survival (31, 32). We hypothesize that inaccurate anchors could delay reporting, but the cited studies did not evaluate short-form video exposure. MRONJ prevention likewise depends heavily on dental assessment and necessary treatment before bone-modifying therapy (12). Among patients with bone metastases, tooth extraction after treatment initiation was an independent risk factor for MRONJ (HR 4.26, 95% CI 2.38–7.44) (10). We hypothesize that risk underestimation could reduce uptake of pretreatment assessment, whereas risk exaggeration could contribute to nonadherence or unplanned care. Evidence on ICI discontinuation and acute-care use (16–18) establishes the relevance of these outcomes but not a causal link to algorithmic exposure. Thus, the proposed exposure-anchoring-SDM-safety sequence remains an untested explanatory model.

Testing this model requires overcoming three obstacles. First, initial consultations, admission assessments, and informed-consent workflows rarely include a standardized short-form video exposure history, leaving exposure largely unobserved. Second, commonly used SDM measures can show ceiling effects (30) and do not directly quantify cognitive anchors. Third, longitudinal studies have not yet connected exposure, anchoring, SDM quality, and clinical safety outcomes within a shared temporal design (19, 20). These gaps prevent reliable estimation of the proposed associations and should be treated as research problems rather than as evidence that the causal chain already exists.

## 3 Discussion

The three DECA modules are proposed responses to the measurement, calibration, and longitudinal-design gaps described above. They are intended to operate sequentially within clinical workflows: DECA-E records exposure, DECA-C evaluates and potentially calibrates anchors, and DECA-L tests longitudinal associations. This is a framework for feasibility and validation studies, not an intervention evaluated in the present Perspective.

DECA-E (Digital Exposure) draws on brief, itemized screening approaches used in national health-information surveillance and tools such as AUDIT-C (33). We propose four fields for the first-visit EMR: frequency of exposure during the previous 30 days to short-form videos about bone tumors or immunotherapy (5-point Likert scale); primary platform (Douyin, Kuaishou, Bilibili, WeChat Channels, or other); self-reported source type (professional organization, verified health professional, patient or peer, or uncertain); and whether family members or primary caregivers were exposed to similar content (21). The module is designed for completion within approximately 2 minutes, but that duration and its measurement performance require prospective testing. Implementation also faces substantial barriers: EMR modification requires institutional and information-technology approval; development and maintenance entail costs; added questions may disrupt high-throughput clinics; and digital infrastructure varies across health systems. A staged implementation is therefore more realistic than universal full integration. Resource-limited sites could begin with a single exposure-frequency item that supports basic stratification, while adding the remaining fields when local capacity permits.

DECA-C (Cognition Calibration) draws conceptually on the Ask-Share-Know model (28) and psychological inoculation against manipulative narratives (34). We propose a two-stage process. A universal screen lasting less than 90 s would assess efficacy expectations (for example, the expected probability that immunotherapy will eliminate the tumor), risk expectations (for example, whether adverse effects are believed to be more severe than those of chemotherapy), and physician-authority expectations (for example, beliefs that physicians favor more expensive drugs). Only patients who screen positive would receive a five-minute calibration script that identifies the narrative technique, offers an evidence-consistent alternative explanation, and then proceeds to formal SDM (26, 27). The five-minute script is not intended for every initial consultation. Trained nursing or pharmacy staff could deliver it under clinician oversight, consistent with task-sharing approaches in oncology care (35). This design may reduce, but will not eliminate, staffing, training, supervision, and workflow burdens. Because DECA-E and the DECA-C screen would both run universally, together they would add approximately 3 minutes to every initial consultation; this cumulative increment, not either module alone, is the figure relevant to clinic-flow planning and should be measured directly in pilot testing. Pilot studies should assess completion time, staff fidelity, acceptability, and effects on clinic flow before scale-up; the oncology-specific effectiveness of psychological inoculation also remains to be established.

DECA-L (Longitudinal Causal Monitoring) is a proposed prospective longitudinal study design, not an analysis conducted in this Perspective. It draws on evidence from ePRO monitoring (31, 32), health-information surveillance infrastructure (33), and causal mediation methods (36–38). From the initial consultation, DECA-E could record short-form video exposure monthly, while DECA-C could measure efficacy, risk, and physician-authority anchors at follow-up visits. SDM-Q-9, CollaboRATE, and the Decision Regret Scale could be administered at treatment initiation, treatment changes, and adverse-event decisions. Prespecified clinical outcomes would include irAE reporting time, completion of MRONJ pretreatment, ICI adherence, and the number of 90-day unplanned visits. Mediation analysis could then estimate direct and indirect associations across exposure, anchoring, SDM quality, and clinical outcomes only under a prespecified temporal structure, adequate control of confounding, and defensible mediation assumptions (36–38). No sample size can be specified responsibly without a formal analysis. It should be determined from expected path effects, the repeated-measures structure, anticipated attrition, and simulation-based power for indirect effects. Longitudinal data alone would not establish causality if these assumptions are not met.

## 4 Conclusion

Current SDM models and PtDAs do not explicitly assess algorithmically reinforced beliefs formed before consultation. Short-form video exposure may contribute to anchoring across efficacy expectations, risk perceptions, and views of physician authority, but its downstream clinical effects have not been established. The proposed mechanism may apply broadly across oncology. Bone tumor and bone metastasis care is presented as a sentinel context because some patients receive both ICIs and bone-modifying agents and because MRONJ prevention includes a time-sensitive pretreatment dental window. We therefore propose moving the operational starting point of SDM upstream to include the patient’s pre-consultation information environment. DECA offers a testable framework for observing exposure, assessing and potentially calibrating anchors, and estimating longitudinal associations with SDM and safety outcomes. Its feasibility, validity, effectiveness, and causal assumptions require prospective evaluation before clinical or policy adoption.

## Data Availability

The original contributions presented in the study are included in the article/supplementary material, further inquiries can be directed to the corresponding authors.
